# Aldosterone and glomerular filtration – observations in the general population

**DOI:** 10.1186/1471-2369-15-44

**Published:** 2014-03-10

**Authors:** Anke Hannemann, Rainer Rettig, Kathleen Dittmann, Henry Völzke, Karlhans Endlich, Matthias Nauck, Henri Wallaschofski

**Affiliations:** 1 Institute of Clinical Chemistry and Laboratory Medicine, University Medicine Greifswald, Greifswald, Germany; 2 Institute of Physiology, University Medicine Greifswald, Greifswald-Karlsburg, Germany; 3 Institute for Community Medicine, University Medicine Greifswald, Greifswald, Germany; 4 Institute of Anatomy and Cell Biology, University Medicine Greifswald, Greifswald, Germany

**Keywords:** Aldosterone, Aldosteronism, Epidemiology, Glomerular Filtration, Renal function

## Abstract

**Background:**

Increasing evidence suggests that aldosterone promotes renal damage. Since data on the association between aldosterone and renal function in the general population are sparse, we chose to address this issue. We investigated the associations between the plasma aldosterone concentration (PAC) or the aldosterone-to-renin ratio (ARR) and the estimated glomerular filtration rate (eGFR) in a sample of adult men and women from Northeast Germany.

**Methods:**

A study population of 1921 adult men and women who participated in the first follow-up of the Study of Health in Pomerania was selected. None of the subjects used drugs that alter PAC or ARR. The eGFR was calculated according to the four-variable Modification of Diet in Renal Disease formula. Chronic kidney disease (CKD) was defined as an eGFR <60 ml/min/1.73 m^2^.

**Results:**

Linear regression models, adjusted for sex, age, waist circumference, diabetes mellitus, smoking status, systolic and diastolic blood pressures, serum triglyceride concentrations and time of blood sampling revealed inverse associations of PAC or ARR with eGFR (ß-coefficient for log-transformed PAC −3.12, p < 0.001; ß-coefficient for log-transformed ARR −3.36, p < 0.001). Logistic regression models revealed increased odds for CKD with increasing PAC (odds ratio for a one standard deviation increase in PAC: 1.35, 95% confidence interval: 1.06-1.71). There was no statistically significant association between ARR and CKD.

**Conclusion:**

Our study demonstrates that PAC and ARR are inversely associated with the glomerular filtration rate in the general population.

## Background

The prevalence of chronic kidney disease (CKD) increases worldwide [1,2]. An impaired renal function is associated with an increased risk of cardiovascular disease and mortality [3,4]. In a large longitudinal study [5] including more than one million subjects from the San Francisco Bay area, a graded association between the eGFR and the risk of cardiovascular events and death was detected. The tight connection between CKD and cardiovascular risk asks for early detection and monitoring of CKD patients.

CKD and cardiovascular diseases share common risk factors, such as age, obesity, diabetes mellitus, smoking, hypertension and dyslipidemia [6]. Increasing evidence suggests that the renin-angiotensin-aldosterone system (RAAS) is involved in linking the metabolic syndrome, CKD and cardiovascular disease [7,8]. Aldosterone, the final product of the RAAS, plays a major role in the regulation of intraglomerular and systemic blood pressure [9]. Primary aldosteronism, a condition characterized by excessive and largely autonomous aldosterone secretion, is associated with a high prevalence of renal damage [10-12]. The deleterious effects of prolonged aldosteronism on the kidney include functional changes (glomerular hyperfiltration) followed by structural changes that induce glomerular ischemia and renal insufficiency [12]. The treatment of patients with primary aldosteronism with aldosterone antagonists such as spironolactone or eplerenone or with adrenalectomy may reduce renal damage and prevent cardiovascular events [13].

Beyond the deleterious cardiovascular and renal effects of excessive circulating aldosterone concentrations [10], the hormone may also play a role in the development of renal disease in the general population not suffering from primary aldosteronism. Since data on the association between aldosterone and renal function in the general population are sparse [14-16], we chose to address this issue. We investigated the associations between the plasma aldosterone concentration (PAC), or the aldosterone-to-renin ratio (ARR) and the estimated glomerular filtration rate (eGFR) in a sample of adult men and women from Northeast Germany.

## Methods

### The study of health in Pomerania (SHIP)

SHIP is a population-based cohort study in the northeast of Germany. Study design and sampling methods have been previously described [17]. In short, 4308 adult men and women between 20–79 years of age participated in the baseline examinations (SHIP-0) between October 1997 and May 2001. The first follow-up examination (SHIP-1) was conducted five years later with 3300 participants being re-examined. The present analyses are based on SHIP-1 data. SHIP is reviewed by an external scientific review board. All participants gave written informed consent. The study conformed to the principles of the Declaration of Helsinki as reflected by an a priori approval of the Ethics Committee of the Board of Physicians Mecklenburg-West Pomerania at the University of Greifswald.

### Instruments and measurements

Socio-demographic characteristics and medical histories of the SHIP-1 participants were obtained by computer-aided personal interviews. Medication was classified using the Anatomical Therapeutic Chemical Classification System (ATC) code. Height, weight, and waist circumference were measured following a standardized protocol. Body mass index (BMI) was calculated as weight (kg)/height^2^ (m^2^). Diabetes mellitus was defined as self-reported physician’s diagnosis or intake of anti-diabetic medication. Subjects were classified in current smokers and non-smokers based on self-report. Systolic and diastolic blood pressures were measured three times on the right arm of seated subjects, using a digital blood pressure monitor (HEM-705CP, Omron Corporation, Tokyo, Japan). The mean of the second and third measurements was used for statistical analyses. Hypertension was defined as systolic blood pressure ≥140 mmHg or diastolic blood pressure ≥90 mmHg or self-reported intake of antihypertensive medication.

Non-fasting blood samples were taken from the cubital vein of subjects in the supine position between 8:30 a.m. and 7:00 p.m. Serum aliquots were stored at −80°C. Serum triglyceride concentrations were determined enzymatically on a Hitachi 717. PAC and PRC were measured in EDTA plasma (PAC: Coat-A-Count Aldosterone, Siemens Healthcare Diagnostics, Eschborn, Germany; PRC: Renin III Generation, Cisbio Bioassay, Bagnols-sur-Cèze Cedex, France). Inter- and intra-assay coefficients of variation of the PAC assay were 15.7% and 5.4% for low and 3.8% and 2.3% for high concentrations, respectively. Inter- and intra-assay coefficients of variation of the PRC assay were 5.0% and 3.6% for low and 4.0% and 0.9% for high concentrations, respectively. The ARR was calculated as the ratio of PAC and PRC. Serum creatinine levels were determined with a modified kinetic Jaffé method (Siemens Dimension RxL; Siemens Healthcare Diagnostics, Eschborn, Germany). The eGFR was calculated according to the four-variable Modification of Diet in Renal Disease formula [18]. CKD was defined as an eGFR <60 ml/min/1.73 m^2^ consistent with the definition of CKD ≥ stage 3 proposed by the National Kidney Foundation Kidney Disease Outcomes Quality Initiative (KDOQI) [19].

### Study population

Of the 3300 SHIP-1 participants, all subjects with missing data on PAC, ARR, or eGFR were excluded from the study (n = 37). Furthermore, all subjects reporting the intake of medication that alters PAC or ARR [diuretics, including aldosterone antagonists, beta blockers or other antiadrenergic agents, calcium channel blockers, angiotensin-converting enzyme (ACE) inhibitors or angiotensin receptor blockers (ARB)] were excluded (n = 1319). Finally, all subjects with missing information on waist circumference, diabetes mellitus, smoking status, all pregnant women, and all subjects with missing data on serum triglyceride concentrations were excluded (n = 23). This resulted in a final study population of 1921 subjects (1020 women).

### Statistical analyses

We report clinical characteristics of the study population as proportions for categorical data and as median (1^st^-3^rd^ quartile) for continuous data. Group comparisons were performed with *χ*^2^-test (categorical data) or Kruskal-Wallis test (continuous data). The associations between PAC or ARR with eGFR were assessed by multivariable analyses of variance and linear regression models. We report adjusted mean eGFR levels with 95% confidence intervals (CI) according to sex-specific tertiles of PAC or ARR. Moreover, we report ß-coefficients with standard errors and p-values from the linear regression models. Due to their skewed distributions, PAC and ARR were log-transformed before being entered in the linear regression models. To assess the associations between PAC or ARR and CKD, multivariable logistic regression models were calculated. Odds ratios (OR) and 95% CI for CKD associated with a one standard deviation (SD) increase in PAC or ARR are reported. Analyses of variance, linear as well as logistic regression models were adjusted for sex, age, waist circumference, diabetes mellitus, smoking status, systolic and diastolic blood pressures, serum triglyceride concentrations, and time of blood sampling (before 10.00 a.m., 10.00-11.59 a.m., 12.00-1.59 p.m., 2.00-3.59 p.m., after 4.00 p.m.). In a sensitivity analysis the models with PAC as independent variable were additionally adjusted for PRC. P-values <0.05 were considered statistically significant. All statistical analyses were performed with SAS 9.1 (SAS Institute Inc., Cary, NC, USA).

## Results

Our study population comprised 1921 participants, including 1020 women (53.1%). The study participants had a median age of 46.0 years (1^st^-3^rd^ quartile: 37.0-58.0 years) and a mean eGFR of 88.1 ml/min/1.73 m^2^ (1^st^-3^rd^ quartile: 77.2-100.4 ml/min/1.73 m^2^). Hypertension was found in 549 subjects (28.6%). CKD was detected in 74 subjects (3.9%), while 878 subjects (45.7%) had an eGFR >90 ml/min/1.73 m^2^. The characteristics of the study population according to sex-specific PAC or ARR tertiles are presented in Table 1.

**Table 1:** Characteristics of the study population

**Characteristics**	**PAC**			**ARR**		
	**1. Tertile**	**2. Tertile**	**3. Tertile**	**1. Tertile**	**2. Tertile**	**3. Tertile**
Men, %	46.2	48.1	46.5	47.0	46.8	46.9
Age, years	48 (38–60)	46 (37–59)	44 (36–55)†,‡	45 (37–56)	45 (35–57)	48 (39–60)†,‡
Current smokers, %	30.2	34.1	37.9†	35.9	34.7	32.0
Diabetes mellitus, %	3.7	2.4	3.5	3.6	2.8	3.1
Waist circumference, cm	87.2 (78.1-96.5)	87.5 (79.0-97.0)	89.0 (79.0-99.0)†	87.0 (78.1-96.5)	87.1 (78.5-97.0)	90.1 (80.1-98.8)†,‡
Systolic BP, mmHg	126.0 (115.0-137.5)	127.0 (115.5-139.0)	126.0 (115.5-138.0)	123.5 (114.0-136.0)	126.0 (115.0-137.0)	129.5 (117.0-140.5)†,‡
Diastolic BP mmHg	80.0 (74.0-86.5)	80.5 (74.5-87.5)	81.0 (74.5-89.0)†	79.0 (73.5-85.3)	80.0 (74.0-87.0)	82.5 (76.0-89.5)†,‡
Triglycerides, mmol/l	1.24 (0.83-1.93)	1.35 (0.92-2.04)*	1.36 (0.90-2.02)†	1.27 (0.85-1.93)	1.27 (0.86-1.96)	1.40 (0.95-2.14)†,‡
PAC, ng/l	21.0 (14.0-26.0)	42.0 (36.0-48.0)*	75.0 (61.0-98.0)†,‡	23.0 (15.0-36.0)	44.0 (32.0-59.0)*	61.0 (45.0-88.0)†,‡
PRC, ng/l	6.6 (4.4-9.8)	7.6 (5.0-11.0)*	9.3 (6.2-14.3)†,‡	10.3 (6.9-14.8)	8.4 (5.9-11.6)*	5.5 (3.8-7.8)†,‡
ARR	2.7 (1.8-4.3)	5.4 (3.8-8.2)*	8.4 (5.8-12.6)†,‡	2.5 (1.8-3.1)	5.2 (4.5-6.3)*	10.5 (8.5-14.2)†,‡
eGFR, ml/min/1.73 m^2^	89.1 (78.3-101.9)	88.0 (76.6-101.1)	87.5 (76.8-99.2)†	91.1 (79.1-104.3)	89.8 (77.9-101.1)	84.5 (75.5-95.8)†,‡

ARR, aldosterone-to-renin ratio; BMI, body mass index; BP, blood pressure; eGFR, estimated glomerular filtration rate; PAC, plasma aldosterone concentration; PRC, plasma renin concentration.

Data are presented as median (1^st^-3^rd^ quartile) for continuous variables and as proportion for categorical variables.

*p < 0.05, 1^st^ vs. 2^nd^ tertile; †p < 0.05 1^st^ vs. 3^rd^ tertile; ‡p < 0.05 2^nd^ vs. 3^rd^ tertile. Group differences were tested with *χ*^2^tests (categorical variables) or Kruskal-Wallis tests (continuous variables).

**Figure 1:**
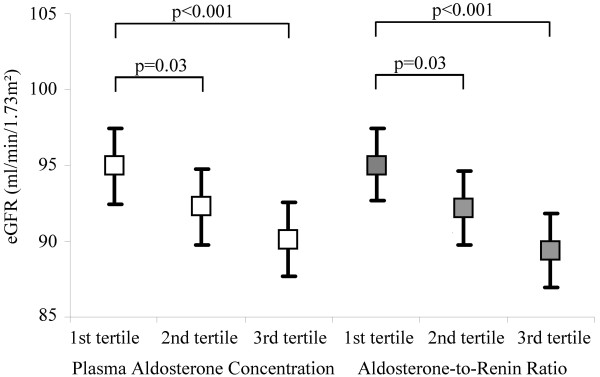
**Adjusted mean eGFR according to sex-specific tertiles of PAC or ARR in 1921 SHIP-1 participants.** PAC, plasma aldosterone concentration; ARR, aldosterone-to-renin ratio. The multivariable analysis of variance models were adjusted for age, sex, waist circumference, systolic and diastolic blood pressures, diabetes mellitus, smoking status, serum triglyceride concentrations, time of blood sampling, and additionally plasma renin concentration in models with PAC or ARR as independent variables. The estimated glomerular filtration rate (eGFR) was calculated according to the four-variable Modification of Diet in Renal Diseases formula.

In analyses of variance models, PAC and ARR but not PRC were inversely associated with eGFR (Figure 1). The adjusted mean eGFR was significantly lower in subjects with PAC or ARR in the second or third tertile than in subjects with PAC or ARR in the first tertile. Linear regression models confirmed the inverse associations between PAC or ARR and renal function. The eGFR decreased with increasing PAC or ARR (ß-coefficient for log-transformed PAC −3.12, standard error 0.56, p < 0.001; ß-coefficient for log-transformed ARR −3.36, standard error 0.49, p < 0.001). The decrease in eGFR with increasing PAC was even more pronounced (ß-coefficient for log-transformed PAC −3.73, standard error 0.58, p < 0.001) after additional adjustment for PRC.

Logistic regression models revealed that increasing PAC was associated with rising odds for CKD (OR for a one SD increase in PAC: 1.35, 95% CI 1.06-1.71). The additional adjustment for PRC hardly affected the model (OR for a one SD increase in PAC: 1.34, 95% CI 1.05-1.70). There were no statistically significant associations between ARR and CKD (OR for a one SD increase in ARR: 1.03, 95% CI 0.89-1.20).

## Discussion

The main finding of our study is that PAC and ARR are inversely associated with the eGFR in the general adult population. These associations were independent of major risk factors for cardiovascular and renal damage, including age, sex, diabetes mellitus, smoking status, waist circumference, systolic and diastolic blood pressures, and serum triglyceride concentrations.

In recent years, experimental and clinical but also observational studies demonstrated a pathogenic role for aldosterone in renal damage [9,20]. Studies in patients with primary aldosteronism revealed a high prevalence of renal damage [21,22] and higher rates of albuminuria or proteinuria than in patients with essential hypertension [10,21,23]. Moreover, in patients with primary aldosteronism serum aldosterone and potassium concentrations are important predictors of renal impairment [24]. In primary aldosteronism, the adrenal aldosterone secretion is partly autonomous from the RAAS [25]. Patients with primary aldosteronism present with high circulating aldosterone and suppressed renin concentrations and elevated ARRs. Our data provide important new information on the relation between the RAAS and renal function by extending previous clinical observations in patients to the general population. Specifically, our data show that high PAC and high ARR are associated with decreasing eGFR. As it is unlikely that a significant proportion of the study population had primary aldosteronism, the present study suggests, that high circulating aldosterone levels within the normal range may have a negative impact on renal function.

Studies investigating the associations between components of the RAAS and renal function in the general population have been rare and produced conflicting results [14-16]. One large cross-sectional study [16], including 9495 outpatient U.S. adults, examined the associations between PAC, plasma renin activity (PRA) or ARR with CKD. This study [16] showed that high PAC (fourth vs. first quartile) or PRA (second to fourth vs. first quartile) were independently associated with CKD ≥ stages 3 (eGFR <60 vs. ≥60 ml/min/1.73 m^2^) and 4 (eGFR <30 vs. ≥60 ml/min/1.73 m^2^). Furthermore, subjects with a high ARR (fourth vs. first quartile) had a decreased OR for CKD ≥ stages 3 and 4. While the ARR was not significantly associated with CKD in the present study, we did find an inverse association between the ARR and eGFR. The different study results with respect to the association between ARR and CKD may be due to the fact that our study population comprised substantially more healthy subjects than the U.S. study population, e.g. the proportions of hypertension or diabetes mellitus were 28.6% and 3.2%, respectively, in SHIP and 83.1% and 25.8%, respectively, in the U.S. sample [16]. Moreover, there were only few subjects with CKD in our study sample (3.9%), which may explain why we did not find an association between ARR and CKD.

Two recent longitudinal studies [14,15] provided evidence for an association between components of the RAAS and incident CKD. The Framingham Offspring Study [14] detected a positive association between the serum aldosterone concentration and incident CKD. In this study [14], 2345 participants free of CKD were followed over a mean duration of 9.5 years. In a recent Japanese study [15] 689 men and women were followed over a median duration of 9.7 years. There was no association between PAC and incident CKD [15] but an inverse association between ARR and incident CKD [15].

Aldosterone may contribute to renal impairment via direct and indirect effects. Direct effects are probably mediated through the mineralocorticoid receptor by causing tubulointerstitial inflammation and subsequent fibrosis [26,27]. In an experimental study [28], transgenic rats with increased RAAS activity demonstrated albuminuria and podocyte damage, which was improved by treatment with the mineralocorticoid receptor antagonist spironolactone. Further animal studies [29-31] revealed a role for aldosterone in the development of glomerulosclerosis and proteinuria through thrombotic and proliferative lesions in the glomeruli and renal vessels. Clinical studies including patients with primary aldosteronism support the hypothesis of a direct effect of aldosterone on renal glomerular filtration in human patients [21,23]. It was shown [23], that patients with primary aldosteronism had a higher urinary albumin excretion than patients with essential hypertension that were matched for mean arterial pressure. In individuals without primary aldosteronism the situation is less clear. In patients with heart failure [32] or resistant hypertension [33] aldosterone antagonists were associated with initial decreases in eGFR and did not alter the decline in renal glomerular function in the long-term. On the other side, there is evidence, that aldosterone antagonists exert renoprotective effects through reduction of albuminuria or proteinuria [26,34,35]. The addition of aldosterone antagonists vs. placebo to ACE inhibitor treatment was associated with a decrease of albuminuria or proteinuria in six double-blind randomized placebo-controlled clinical trials [36-41], reviewed in 2010 [26]. Yet, in four [36,38-40] out of the six studies, the decreases in albuminuria and proteinuria were paralleled by decreases in blood pressure. Direct aldosterone-induced effects on renal glomerular filtration may thus be limited in comparison to the impact of indirect effects of the hormone on the kidney, which are exerted through increases in systemic and intraglomerular blood pressure. It is well known, that high circulating aldosterone concentrations, even within the physiological range, predispose persons to an increased risk of hypertension [42]. High systolic blood pressure, in turn, results in an increased rate of renal function loss [43]. In the present study, we observed inverse associations between PAC or ARR and eGFR despite adjusting for systolic and diastolic blood pressures in the regression models, suggesting that aldosterone may have affected eGFR through blood pressure-independent mechanisms. Thus, our data argues for a direct relation between the measures. Moreover, the relation between PAC and eGFR may be bidirectional. As kidney function decreases, renal potassium excretion is impaired. The resulting increase in plasma potassium concentrations in turn may stimulate aldosterone secretion [44].

Overall, our results provide evidence for an inverse association of PAC or ARR with eGFR in the general population free of medication that alters PAC or ARR. The impact of PAC or ARR on eGFR in our sample of adults with mainly normal renal function was statistically significant but rather small. Clinical implications of decreasing eGFR with higher PAC or ARR may therefore be marginal. On the other hand, the association between PAC or ARR and eGFR is of importance as even a moderately reduced kidney function increases the risk for cardiovascular disease and mortality [3,6,45].

The present study has several strengths and limitations. Strengths include the population-based design, the extensive characterization of the SHIP participants and the standardized data collection by trained and certified examiners. Limitations arise from the cross-sectional study design, which does not allow analyzing causality between the measures. Thus, we cannot assess whether it is the high PAC that causes the decrease in eGFR or whether it is the decreased eGFR that leads to the increase in PAC. It might also be questioned whether a single–occasion measurement of PAC or PRC can appropriately represent the participants’ hormonal status. Unfortunately, the epidemiological study design did not allow us to perform repeated blood samplings and hormone measurements. All our data were obtained from Caucasian subjects. The transferability of our results to other ethnicities may thus be limited.

## Conclusion

In conclusion, our study demonstrates that circulating aldosterone is inversely associated with the glomerular filtration rate in the general population. Further studies are warranted to assess the associations between aldosterone and CKD in the general population but also to develop strategies to identify patients who might benefit from treatment with aldosterone antagonists.

## Competing interests

The authors declare that there is no conflict of interests that could be perceived as prejudicing the impartiality of the research reported.

## Authors’ contributions

Study design, data analysis, and data interpretation: AH, RR, KD, HV, KE, MN, HW. Drafting manuscript and revising manuscript content: AH, RR, KD, HV, KE, MN, HW. Approving final version of manuscript: AH, RR, KD, HV, KE, MN, HW. All authors read and approved the final manuscript.

## Pre-publication history

The pre-publication history for this paper can be accessed here:

http://www.biomedcentral.com/1471-2369/15/44/prepub
